# Soothing and Healing

**Published:** 1875-12

**Authors:** 


### SOOTHING AND HEALING.

These are the characteristics of Mr. Packer's two products known as "Packer's Tar Soap" and "Packer's Charm," as stated by scores of persons who have used them at our suggestion. Perhaps these words cover the whole subject, but we doubt it. There certainly is something more potent in the wonderful efficacy manifested by these preparations in unwholesome looking eruptions, and irritations of the skin. Healing and soothing substances, only *protect*, usually. They serve to keep out dust and germs of disease and animal life. But "Packer's Tar Soap" clearly has power to *neutralize* disease and terminate it.

A good physician and friend has suffered from a very unpleasant peeling and cracking of the skin of the hands for a lifetime, and has been cured in a month or two by the Soap and the Charm. He has suffered much, for his hands have rarely been without raw spots in cold weather. It was a blood-taint, no doubt, and "Packer's Tar Soap," aided by "Packer's Charm," has effected a complete cure.

Another friend has found great relief from the use of these two articles in piles. He uses both, each night and each morning, and considers himself cured of this painful disease from which he suffered since 1864.

Dr. H. W. Kitchen, Professor of Anatomy in the Wooster Medical University, deems Packer's Tar Soap the best deoderizer in use for surgeons, and he advises his students to use it for purposes of disinfection after dissections and in all offensive diseases. The dentists are using it with great satisfaction, finding that the odors em anating from ulcerated teeth and carelessly-kept mouths, and the many unclean things which the members of this useful profession have to handle, cannot be as perfectly neutralized in any other way.

These two preparations are great beautifiers, too, as they make the skin soft and smooth, and destroy the worst effects of exposure and hard usage. They are coming into almost universal use in this vicinity and we presume their employment is widely extending everywhere.

---

An English aeronaut lately tested a machine of his own patent called the parakite. It is 30 feet high and 30 feet wide. As soon as the sail was fixed over the framework and the front or windward point of the parakite raised, so as to allow the wind to touch the machine on its under surface, it was instantly converted into a concave form and showed symptoms of rising. The wind was blowing at the rate of not more than two miles an hour, but with this slight breeze the aeronaut was carried into the air. The idea is to put it to practical utility for war purposes, engineering, and signalling, where it is necessary to attain lofty elevations. The machine covers an area of 700 superficial feet, and its entire weight is 100 pounds. The inventor asserts that it can be used successfully in any wind, ranging between four and forty miles an hour, and an altitude of from 600 to 1,000 feet can be attained.

